# Gaboxadol in Fragile X Syndrome: A 12-Week Randomized, Double-Blind, Parallel-Group, Phase 2a Study

**DOI:** 10.3389/fphar.2021.757825

**Published:** 2021-10-08

**Authors:** Dejan B. Budimirovic, Kelli C. Dominick, Lidia V. Gabis, Maxwell Adams, Mathews Adera, Linda Huang, Pamela Ventola, Nicole R. Tartaglia, Elizabeth Berry-Kravis

**Affiliations:** ^1^ Department of Psychiatry, Kennedy Krieger Institute, Johns Hopkins University, Baltimore, MD, United States; ^2^ Department of Psychiatry and Behavioral Sciences-Child Psychiatry, Johns Hopkins School of Medicine, Baltimore, MD, United States; ^3^ Department of Psychiatry, University of Cincinnati College of Medicine, Cincinnati, OH, United States; ^4^ Cincinnati Children’s Hospital Medical Center, Cincinnati, OH, United States; ^5^ Maccabi HMO, Tel Aviv-Yafo, Israel; ^6^ Sackler School of Medicine, Tel Aviv University, Tel Aviv-Yafo, Israel; ^7^ Ovid Therapeutics Inc., New York, NY, United States; ^8^ Child Study Center, Yale University, New Haven, CT, United States; ^9^ University of Colorado School of Medicine, Children’s Hospital Colorado, Aurora, CO, United States; ^10^ Department of Pediatrics, Neurological Sciences, Biochemistry, Rush University Medical Center, Chicago, IL, United States

**Keywords:** OV101, gaboxadol, fragile X syndrome, *FMR1*, GABA_A_, safety, efficacy, randomized study

## Abstract

**Background:** Fragile X syndrome (FXS), the most common single-gene cause of intellectual disability and autism spectrum disorder (ASD), is caused by a >200-trinucleotide repeat expansion in the 5’ untranslated region of the fragile X mental retardation 1 (*FMR1*) gene. Individuals with FXS can present with a range of neurobehavioral impairments including, but not limited to: cognitive, language, and adaptive deficits; ASD; anxiety; social withdrawal and avoidance; and aggression. Decreased expression of the γ-aminobutyric acid type A (GABA_A_) receptor *δ* subunit and deficient GABAergic tonic inhibition could be associated with symptoms of FXS. Gaboxadol (OV101) is a *δ*-subunit–selective, extrasynaptic GABA_A_ receptor agonist that enhances GABAergic tonic inhibition, providing the rationale for assessment of OV101 as a potential targeted treatment of FXS. No drug is approved in the United States for the treatment of FXS.

**Methods:** This 12-weeks, randomized (1:1:1), double-blind, parallel-group, phase 2a study was designed to assess the safety, tolerability, efficacy, and optimal daily dose of OV101 5 mg [once (QD), twice (BID), or three-times daily (TID)] when administered for 12 weeks to adolescent and adult men with FXS. Safety was the primary study objective, with key assessments including treatment-emergent adverse events (TEAEs), treatment-related adverse events leading to study discontinuation, and serious adverse events (SAEs). The secondary study objective was to evaluate the effect of OV101 on a variety of problem behaviors.

**Results:** A total of 23 participants with FXS (13 adolescents, 10 adults) with moderate-to-severe neurobehavioral phenotypes (Full Scale Intelligence Quotient, 41.5 ± 3.29; ASD, 82.6%) were randomized to OV101 5 mg QD (*n* = 8), 5 mg BID (*n* = 8), or 5 mg TID (*n* = 7) for 12 weeks. OV101 was well tolerated across all 3 treatment regimens. The most common TEAEs were upper respiratory tract infection (*n* = 4), headache (*n* = 3), diarrhea (*n* = 2), and irritability (*n* = 2). No SAEs were reported. Improvements from baseline to end-of-treatment were observed on several efficacy endpoints, and 60% of participants were identified as treatment responders based on Clinical Global Impressions-Improvement.

**Conclusions:** Overall, OV101 was safe and well tolerated. Efficacy results demonstrate an initial signal for OV101 in individuals with FXS. These results need to be confirmed in a larger, randomized, placebo-controlled study with optimal outcomes and in the most appropriate age group.

**Clinical Trial Registration:** www.ClinicalTrials.gov, identifier: NCT03697161

## Introduction

Fragile X syndrome (FXS) is an inherited neurodevelopmental disorder caused by a full-mutation expansion [>200 trinucleotide (CGG) repeats] in the promoter region of the fragile X mental retardation 1 (*FMR1*) gene. The resulting epigenetic silencing of *FMR1* causes a deficiency in or absence of the gene’s encoded protein, fragile X mental retardation protein (FMRP) (Bagni et al., 2012). With an estimated prevalence of approximately 1 in 4,000 males and 1 in 6,000 females, FXS is the most common single-gene cause of intellectual disability (ID) and autism spectrum disorder (ASD) (Crawford et al., 2001; Tassone et al., 2012). Individuals with FXS can present with a broad range of neurobehavioral abnormalities, including cognitive deficits e.g., ID, 95% of males, 35% of females (Rousseau et al., 1994; Wright-Talamante et al., 1996); language disorders (non-verbal to perseverative); ASD (51% of males); psychiatric and behavioral impairments, such as anxiety, hyperarousal, and other sensory processing difficulties; repetitive behaviors; attentional network deficits; and irritability often accompanied by aggressive and/or self-injurious behaviors (Hagerman et al., 2009; Kaufmann et al., 2017; Raspa et al., 2018; Budimirovic et al., 2020; Reisinger et al., 2020). Patients with FXS also present with neurological abnormalities, such as motor and coordination difficulties (Gabis et al., 2011) and a higher incidence of epilepsy (Hagerman et al., 2009). As an X-linked disorder, males with FXS have a more severe phenotype than females, with evidence suggesting an inverse relationship between FMRP deficiency and severity of FXS-associated neurobehavioral phenotype (Kim et al., 2019; Budimirovic et al., 2020). FXS phenotype severity can also be affected by size mosaicism (premutation), X-chromosome inactivation in females, and variation in the methylation status of full mutations (Nolin et al., 1994; Budimirovic et al., 2020).

Symptom-based, off-label treatments used in the management of patients with FXS include psychostimulants for attention deficit hyperactivity disorder (ADHD) symptoms; α_2_-adrenergic receptor agonists for sensory overstimulation, hyperarousal, hyperactivity, and sleep disturbances; anticonvulsants for seizures and mood instability; selective serotonin reuptake inhibitors for anxiety; and antipsychotics and antidepressants for aggression, anxiety, and sleep disturbances (Berry-Kravis et al., 2012; Eckert et al., 2019). However, few randomized, controlled studies have been conducted to formally evaluate these symptomatic interventions in FXS (Berry-Kravis et al., 2018). Although safe and effective treatments for FXS are needed, particularly for targeted treatments that surpass symptom-based management, no medication is approved in the United States (Lee et al., 2018).

Evidence suggests that GABAergic dysfunction and the resulting excitatory and inhibitory imbalance can contribute to the pathophysiology of FXS (Berry-Kravis et al., 2018). GABA (γ-aminobutyric acid) is the primary inhibitory neurotransmitter in the brain. *Fmr1* knockout (KO) mice exhibit decreased GABA type A (GABA_A_) receptor *δ* subunit expression, GABA synthesis, and GABA levels, resulting in reduced tonic inhibition. Reduced tonic inhibition may, in turn, lead to abnormal excitatory signaling in the brain, culminating in a range of symptoms (Olmos-Serrano et al., 2011; Zafarullah and Tassone, 2019). Tonic inhibition, mediated by *δ*-subunit–containing GABA_A_ receptors, plays an important role in various functions of different regions in the brain (Cope et al., 2005; Brickley and Mody, 2012; Lee and Maguire, 2014; Whissell et al., 2015) and is a potential treatment target for FXS.

Evidence from clinical studies of GABAergic therapies, specifically arbaclofen (GABA_B_ agonist), ganaxolone (GABA_A_ receptor modulator), and acamprosate (GABA_B_ and GABA_A_ receptor modulator), suggests that GABA receptor modulation may hold therapeutic potential for treating the core behavioral problems associated with FXS (Zafarullah and Tassone, 2019). For example, in 2 placebo-controlled, phase 3 studies of patients with FXS aged 5–11 or 12–50 years, arbaclofen did not differ statistically from placebo on the primary outcome measure of social avoidance on the Aberrant Behavior Checklist-Community Edition (ABC-C) refactored for FXS (ABC-C_FXS_) (Sansone et al., 2012). However, improvements in irritable behavior and parenting stress were observed for children who received the highest drug dose [10 mg three-times daily (TID)] (Berry-Kravis et al., 2017).

Gaboxadol (OV101) is a *δ*-subunit–selective extrasynaptic GABA_A_ receptor agonist that has been shown to be well tolerated and to confer improvements in sleep induction in 2 double-blind, placebo-controlled studies of adults with insomnia (Roth et al., 2010). Mechanistically, in *Fmr1* KO mice, OV101 restored tonic inhibition in the amygdala, reduced sensory hypersensitivity and motor hyperactivity, and improved pre-pulse inhibition (Olmos-Serrano et al., 2011). In other *Fmr1* KO mouse studies, OV101 also normalized hyperactivity and repetitive, social, and anxiety-like behavior, which have been associated with decreased expression of the GABA_A_ receptor *δ* subunit and deficient GABAergic tonic inhibition (Gantois et al., 2006; Olmos-Serrano et al., 2010; Martin et al., 2014). Thus, OV101 may be effective in treating humans with FXS.

Here, we report results from the first interventional clinical study of OV101 in FXS. This 12-weeks, randomized, double-blind, proof-of-concept, phase 2a study evaluated the safety and efficacy profiles of multiple doses of OV101 in adolescent and adult males with FXS.

## Materials and Methods

### Study Participants

Adolescent and adult males aged 13–22 years inclusive who had a clinically- and molecularly-confirmed full mutation of the *FMR1* gene, were moderately-to-severely affected by FXS [score of ≥4 on the Clinical Global Impressions–Severity (CGI-S) scale], and had a Full Scale Intelligence Quotient score <75, were eligible to participate. Antiepileptic and/or psychoactive medication use was permitted if no more than 3 such medications were being used and the dose-regimen for each medication was stable for at least 4 weeks before randomization and then maintained throughout the study.

To ensure that the effects of OV101 were evaluated in a patient population with minimal FMRP expression, females were excluded from the study. Other exclusion criteria included a history of uncontrolled seizure disorder or seizure episodes within 6 months of screening, a change in anticonvulsant pharmacotherapy within 3 months of screening, use of a GABAergic agent on a regular schedule, use of a cannabinoid derivative, a history of suicidal behavior, and any clinically significant medical condition or laboratory finding at screening that could interfere with study conduct/participation or pose an unacceptable risk.

### Study Design

**FIGURE 1:**
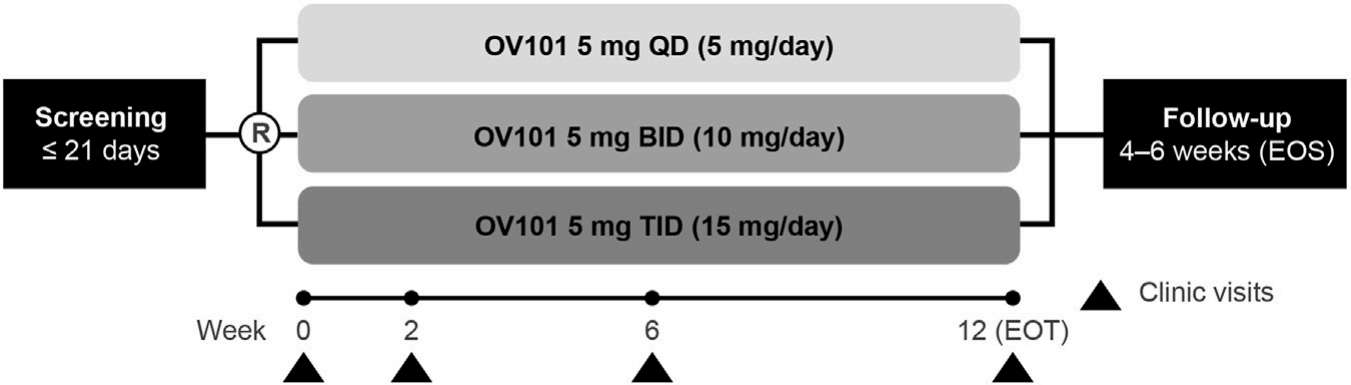
Study design. R, randomization; OV101, gaboxadol; QD, once daily; BID, twice daily; TID, 3-times daily; EOT, end of treatment; EOS, end of study.

This randomized, double-blind, parallel-group, phase 2a study (ClinicalTrials.gov identifier: NCT03697161), conducted at 7 sites in the United States and 1 site in Israel, was designed to assess the safety, tolerability, and efficacy of 3 OV101 dose-regimens administered over 12 weeks to patients with FXS (Figure 1). Independent ethics review boards approved the trial protocol at each trial site. Eligible participants were randomized (1:1:1) to receive OV101 5 mg once daily (QD), twice daily (BID), or TID for 12 weeks. Randomization was stratified by age group (adolescent vs. adult). All patients received study medication TID (morning, afternoon, and evening), with OV101 administered to all participants in the morning and either OV101 or placebo administered in the afternoon and evening throughout the treatment period (including baseline). Dose adjustments were not permitted.

Clinic visits were scheduled at weeks 2, 6, and 12 [end of treatment (EOT)], with an end-of-study (EOS) follow-up visit at some time during weeks 16 and 18. In addition to the assessments performed during every clinic visit, information on adverse events, concomitant medication use, and suicidality were collected during phone calls at the end of weeks 1, 4, 8, and 10. The caregiver or legally acceptable representative (LAR) completed paper sleep diaries on behalf of participants, and participants wore actigraphs (wrist-worn sleep monitors) (Dueck et al., 2020) for 7 days immediately preceding the baseline, week 2, week 6, EOT, and EOS visits.

The study was designed by the sponsor in collaboration with a consortium of experts in FXS and approved by the internal review board at each participating site. Informed consent was provided by a caregiver or LAR, and, to the extent possible, participants also assented to the protocol.

### Objectives and Assessments

The primary objective was to assess the safety and tolerability of OV101 during the 12-weeks treatment period. Safety endpoints were treatment-emergent adverse events (TEAEs), treatment-related adverse events, TEAEs leading to study discontinuation, and serious adverse events (SAEs). Rate of OV101 compliance was calculated as: (actual dosage of OV101/expected dosage of OV101) × 100%; patients achieving a rate of ≥80% based on a medication diary maintained by the LAR was considered compliant.

The secondary objective was to evaluate the efficacy of OV101 during the 12-weeks treatment period. Efficacy endpoints were Clinical Global Impressions-Improvement (CGI-I) scale score and changes from baseline in CGI-S total and subscale scores, ABC-C total and subscale scores (Aman et al., 1985), ABC-C_FXS_ total and subscale scores (Sansone et al., 2012), Anxiety, Depression, and Mood Scales (ADAMS) total and subscale scores (Esbensen et al., 2003), Repetitive Behavior Scale–Revised (RBS-R) total and subscale scores (Lam and Aman, 2007), Short Sensory Profile–2 total and subscale scores, and Conners 3 subscale scores (Conners et al., 1998). The ABC-C is a 58-item questionnaire completed by the LAR/caregiver that assesses a range of behaviors, including irritability, lethargy/social withdrawal, inappropriate speech, hyperactivity, and stereotypic behavior (Aman et al., 1985). Each item is rated on a scale of 0–3 (“not at all a problem” to “the problem is severe in degree”). Compared with the ABC-C, ABC-C_FXS_ includes a new evaluation on social avoidance, modified evaluations of irritability, hyperactivity, lethargy/withdrawal, and stereotypy, and a similar evaluation of inappropriate speech (Sansone et al., 2012). ADAMS, a LAR/caregiver-completed assessment that screens comprehensively for anxiety and depression in persons with ID, is a 28-item questionnaire grouped into 5 subscales that assesses the frequency and severity of manic/hyperactive behavior, depressed mood, social avoidance, general anxiety, and obsessive behavior that are rated on a scale of 0–3 (“not a problem” to “severe problem”) (Esbensen et al., 2003). Also completed by the LAR/caregiver, the RBS-R is a 43-item questionnaire assessing a variety of repetitive behaviors with 6 behavior subscales: stereotyped, ritualistic, self-injurious, compulsive, restricted, and sameness (Lam and Aman, 2007). Behaviors are rated on a scale of 0–3 (“behavior does not occur” to “behavior occurs and is a severe problem”). The Short Sensory Profile-2 (PsychCorp, San Antonio, TX) is a LAR/caregiver-completed, 34-item questionnaire that evaluates sensory processing patterns in the context of home, school, and community-based activities based on a scale of 1–5 (“almost never” to “almost always”) (Simpson et al., 2019). The Conners 3rd Edition (North Tonawanda Multi-Health System, North Tonawanda, NY) was used by a LAR/caregiver to rate ADHD; subscales include Assessment of Validity (positive impression, negative impression, inconsistency index), Content Scales (inattention, hyperactivity/impulsivity, learning problems, executive functioning, defiance/aggression, peer relations), and Diagnostic and Statistical Manual of Mental Disorders, Fourth Edition, Text Revision (DSM-IV-TR) Symptom Scales (i.e., ADHD predominantly inattention type, ADHD predominantly hyperactive-impulsive) (Conners et al., 1998).

Exploratory efficacy endpoints included changes from baseline in Parent Global Impressions–Severity (PGI–S) and Parent Global Impressions–Improvement (PGI–I) scores and clinician-rated changes from baseline in the top 3 concerns identified by caregivers [per a visual analog scale (VAS)] (Adams et al., 2011). The concerns were identified on a per-patient basis and could have derived from any symptom domain related to FXS. The severity of each concern was scored by caregivers using a 10-cm VAS and was based on the number of centimeters from the left margin, with anchors of “not at all severe” (left side of the line, 0 cm) and “very severe” (right side of the line, 10 cm).

### Statistical Methods

The safety analyses were performed on the safety population, which comprised all patients receiving ≥1 dose of study drug. The efficacy analyses were performed on the full-analysis set, which comprised all patients receiving ≥1 dose of study drug and having ≥1 post-baseline efficacy assessment. Participants were analyzed according to the treatment to which they were randomized.

Safety and efficacy outcomes were analyzed using descriptive statistics. To investigate any trends visible in the descriptive analyses, a mixed-effects model for repeated measures (MMRM), with dosing regimen, visit, and age group as fixed effects; age and baseline as covariates (if appropriate); and visit × dosing regimen as an interaction, was used. With an assumed unstructured covariance structure, the least squares mean change from baseline [and 95% confidence interval (CI)] for each dosing regimen, as well as the overall study population, at each post-baseline visit was estimated. The least squares mean difference (and corresponding 95% CI) was estimated for each pairwise comparison among the 3 dosing regimens. In the case of a statistically significant age group main effect (*p* < 0.05) in the MMRM or age effects in the descriptive analyses, the mean change (and corresponding 95% CI) was estimated for each age group separately, as well as the overall study population, by including an additional interaction term (age × week × dosing regimen) in the MMRM. Post-hoc analyses of the changes or percent changes from baseline for CGI-S total and subscale scores, ABC-C_FXS_ total and subscale scores, and ADAMS total and subscale scores, were performed using the parametric Student’s t-test.

## Results

### Patients

**FIGURE 2:**
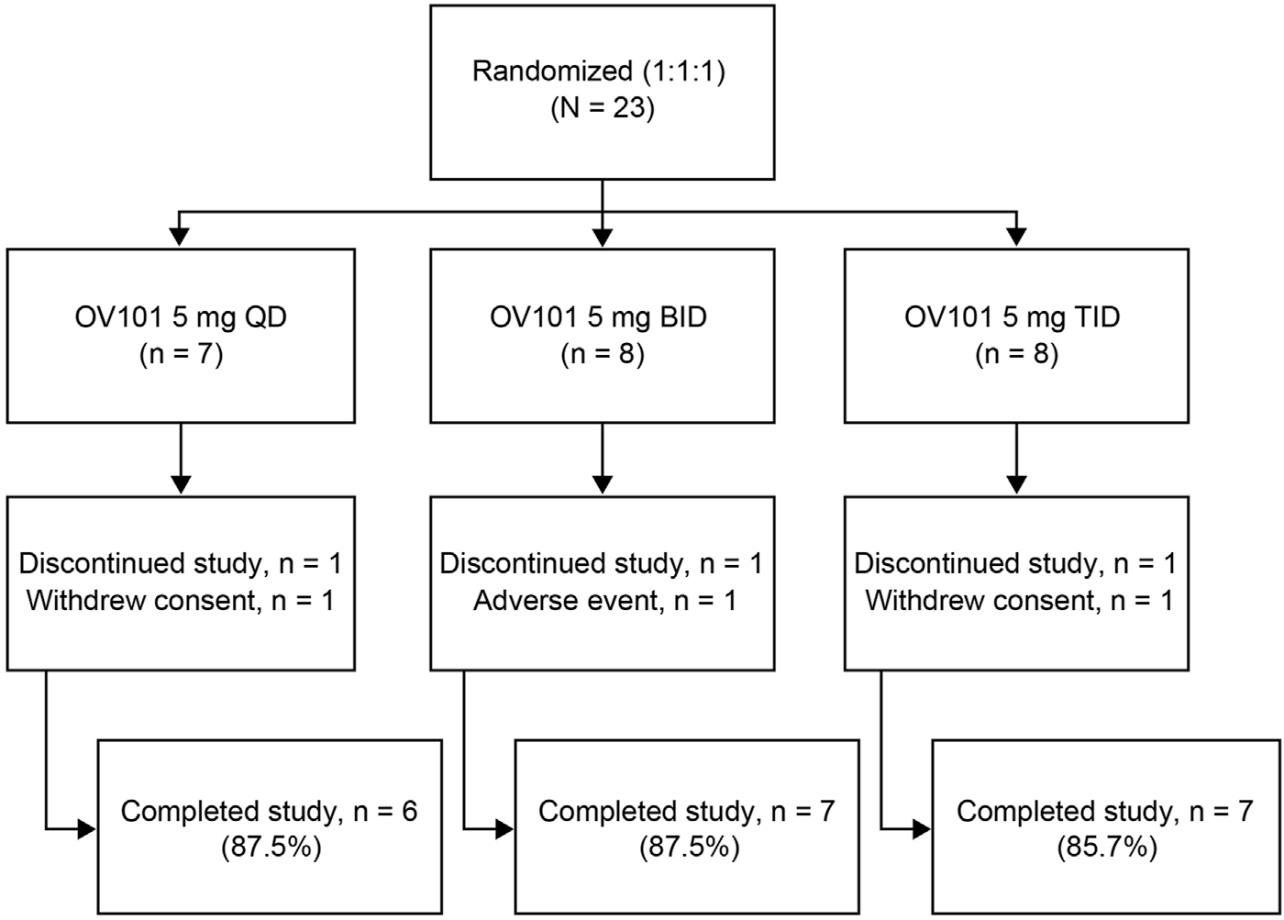
Patient disposition. OV101, gaboxadol; QD, once daily; BID, twice daily; TID, 3-times daily.

A total of 23 participants with FXS (13 adolescents, 10 adults) were randomized to OV101 5 mg QD (*n* = 8), BID (*n* = 8), or TID (*n* = 7) (Figure 2). One participant in each dosing group discontinued the study due to withdrawn consent (QD, *n* = 1; TID, *n* = 1) or TEAE (agitation; BID, *n* = 1).

Baseline demographic and clinical characteristics are summarized in Tables 1, 2, respectively. The overall study population (*n* = 23) exhibited mostly severe neurobehavioral abnormalities, with a mean ± standard deviation (SD) Stanford Binet-5 IQ score (derived according to standard methodology and not z-deviation method) of 41.5 ± 3.29 and verbal IQ score of 44.5 ± 3.23 (Sansone et al., 2014). Of these 23 participants, 82.6% met the Diagnostic and Statistical Manual of Mental Disorders (5th edition) criteria for ASD. The mean rate of OV101 compliance was 95.4% (*n* = 22), with 21 patients considered compliant (≥80% compliance rate) with assigned OV101 treatment.

**TABLE 1:** Baseline demographic characteristics.

Characteristic	OV101 5 mg QD (*n* = 7)	OV101 5 mg BID (*n* = 8)	OV101 5 mg TID (*n* = 8)	Total (*n* = 23)
Male, *n* (%)	7 (100)	8 (100)	8 (100)	23 (100)
Mean age, years (SD)	17.0 (3.46)	16.5 (2.93)	17.5 (3.34)	17.0 (3.12)
Adolescents, *n* (%)^a^	4 (57.1)	5 (62.5)	4 (50.0)	13 (56.5)
Adults, *n* (%)^b^	3 (42.9)	3 (37.5)	4 (50.0)	10 (43.5)
Not Hispanic or Latino, *n* (%)	7 (100)	8 (100)	7 (87.5)	22 (95.7)
Race, *n* (%)				
White	7 (100)	7 (87.5)	6 (75.0)	20 (87.0)
Black or African-American	0	1 (12.5)	0	1 (4.3)
Native Hawaiian or other	0	0	1 (12.5)	1 (4.3)
Pacific Islander				
Other	0	0	1 (12.5)	1 (4.3)
Weight, mean (SD), kg	85.10 (25.03)	76.89 (31.86)	84.16 (35.02)	81.92 (29.97)
Height, mean (SD), cm	171.77 (6.30)	170.39 (10.46)	168.43 (12.72)	170.05 (10.09)

a Ages 13–17 years, inclusive.

b Ages 18–22 years, inclusive.

OV101, gaboxadol; QD, once daily; BID, twice daily; TID, 3-times daily; SD, standard deviation.

**TABLE 2:** Baseline clinical characteristics.

Mean (SD) score	OV101 5 mg QD (*n* = 7)	OV101 5 mg BID (*n* = 8)	OV101 5 mg TID (*n* = 8)	Total (*n* = 23)
FSIQ score	(*n* = 7)	(*n* = 8)	(*n* = 8)	(*n* = 23)
SB-5, full scale standard^a^	41.7 (4.11)	40.5 (0.93)	42.3 (4.10)	41.5 (3.29)
Nonverbal	42.8 (1.79)^b^	42.0 (0.00)	43.6 (3.11)	42.8 (2.14)
Verbal	43.2 (0.45)^b^	44.0 (1.85)	45.8 (4.80)	44.5 (3.23)
Abbreviated	47.6 (1.34)^b^	47.0 (0.00)	48.0 (1.93)	47.5 (1.36)
DSM-5 ASD criteria, *n* (%)	7 (100)	6 (75.0)	6 (75.0)	19 (82.6)
Alcohol use (current or previous), *n* (%)	0 (0)	0 (0)	0 (0)	0 (0)
ABC-C_FXS_	(*n* = 4)	(*n* = 6)	(*n* = 7)	(*n* = 17)
Total score	80.5 (24.93)	60.0 (34.26)	62.1 (18.91)	65.7 (26.26)
Irritability	21.3 (8.42)	20.8 (13.59)	17.1 (11.55)	19.4 (11.18)
Lethargy and social withdrawal	11.3 (9.64)	8.2 (2.86)	11.4 (5.38)	10.2 (5.77)
Inappropriate speech	10.0 (1.83)	6.0 (5.06)	7.4 (2.23)	7.5 (3.59)
Hyperactivity	16.8 (5.06)	12.5 (10.41)	11.1 (6.87)	12.9 (7.84)
Stereotypic behavior	15.0 (3.56)	8.8 (6.31)	10.0 (3.00)	10.8 (4.93)
Social avoidance	6.3 (3.30)	3.7 (3.33)	5.0 (4.12)	4.8 (3.59)
CGI-S (at baseline)	(*n* = 7)	(*n* = 8)	(*n* = 8)	(*n* = 23)
Total score	5.1 (0.90)	4.6 (0.74)	4.6 (0.74)	4.8 (0.80)
Anxiety	4.6 (1.72)	4.3 (0.46)	4.5 (0.76)	4.4 (1.04)
ADHD	3.9 (0.69)	4.4 (0.92)	4.5 (0.93)	4.3 (0.86)
Communication/connectedness	5.1 (0.69)	4.3 (0.71)	4.4 (0.92)	4.6 (0.84)
Repetitive and restrictive behavior	4.9 (1.35)	4.3 (0.89)	4.1 (1.13)	4.4 (1.12)
Disruptive behavior	4.0 (1.63)	4.0 (1.20)	3.8 (1.58)	3.9 (1.41)
Activities of daily living	4.7 (1.89)	4.5 (0.93)	4.6 (1.06)	4.6 (1.27)
ADAMS	(*n* = 7)	(*n* = 8)	(*n* = 8)	(*n* = 23)
Total score	28.4 (17.19)	20.1 (9.00)	26.1 (9.13)	24.7 (12.07)
Manic/hyperactive behavior	7.3 (4.50)	6.4 (4.31)	6.3 (3.65)	6.6 (3.99)
Depressed mood	1.6 (2.15)	2.3 (1.83)	2.3 (2.12)	2.0 (1.97)
Social avoidance	10.4 (5.47)	5.0 (4.00)	7.6 (4.96)	7.6 (5.11)
General anxiety	7.3 (5.06)	5.6 (3.38)	8.0 (3.21)	7.0 (3.87)
Obsessive/compulsive behavior	3.0 (3.92)	1.8 (2.19)	3.1 (1.96)	2.6 (2.71)
RBS-R	(*n* = 7)	(*n* = 8)	(*n* = 8)	(*n* = 23)
Total score	35.3 (31.38)	30.3 (24.14)	26.0 (12.24)	30.3 (22.72)
Conners 3	(*n* = 7)	(*n* = 3)	(*n* = 7)	(*n* = 17)
Inattention	11.1 (3.98)	6.3 (0.58)	10.1 (3.08)	9.9 (3.55)
Hyperactivity/impulsivity	7.7 (5.41)	3.0 (1.00)	6.7 (4.46)	6.5 (4.64)
Short Sensory Profile–2	(*n* = 7)	(*n* = 8)	(*n* = 8)	(*n* = 23)
Total score	83.9 (25.43)	83.1 (36.91)	91.1 (6.96)	86.1 (25.28)
PGI-S	5.1 (1.07)	5.0 (1.20)	5.1 (0.64)	5.1 (0.95)

a Since the SB-5 is not available in Hebrew, patients at the Israel study site used an alternative assessment to yield a full-scale IQ with nonverbal and verbal scores.

b *n* = 5.

SD, standard deviation; FSIQ, full-scale intelligence quotient; SB-5, Stanford-Binet Intelligence Scale, 5th edition; DSM-5, Diagnostic and Statistical Manual of Mental Disorders, 5th edition; ASD, autism spectrum disorder; ABC-C_FX_, Aberrant Behavior Checklist–Community factor score for fragile X syndrome; CGI-S, Clinical Global Impressions–Severity; ADHD, attention deficit hyperactivity disorder; ADAMS, Anxiety, Depression, and Mood Scales; PGI–S, Parent Global Impressions–Severity; OV101, gaboxadol; QD, once daily; BID, twice daily; TID, 3-times daily.

### Safety and Tolerability

Table 3 summarizes safety and tolerability measures for OV101. The mean ± SD duration of exposure to OV101 was 73.1 ± 31.85, 77.1 ± 19.47, and 75.3 ± 25.64 days for the QD, BID, and TID regimens, respectively. The majority of participants (*n* = 16, 69.6%) reported ≥1 TEAE (QD, 28.6%; BID, 100.0%; TID, 75.0%). Across all dosing regimens, the only TEAEs occurring in ≥2 patients were upper respiratory tract infection (*n* = 4, 17.4%), headache (*n* = 3, 13.0%), diarrhea (*n* = 2, 8.7%), and irritability (*n* = 2, 8.7%).

**TABLE 3:** Summary of OV101 safety and tolerability.

Patients, *n* (%)	OV101 5 mg QD (*n* = 7)	OV101 5 mg BID (*n* = 8)	OV101 5 mg TID (*n* = 8)	Total (*n* = 23)
Any TEAE	2 (28.6)	8 (100)	6 (75.0)	16 (69.6)
Severity of most severe TEAE				
Mild	2 (28.6)	7 (87.5)	6 (75.0)	15 (65.2)
Moderate	0	1 (12.5)	0	1 (4.3)
Severe	0	0	0	0
TEAEs occurring in ≥2 participants				
Diarrhea	0	0	2 (25.0)	2 (8.7)
Irritability	0	1 (12.5)	1 (12.5)	2 (8.7)
Headache	0	3 (37.5)	0	3 (13.0)
Upper respiratory tract infection	0	3 (37.5)	1 (12.5)	4 (17.4)
Any treatment-related TEAE	1 (14.3)	5 (62.5)	2 (25.0)	8 (34.8)
Any TRAE leading to early termination	0	1 (12.5)	0	1 (4.3)
Agitation	0	1 (12.5)	0	1 (4.3)
Any SAE	0	0	0	0

OV101, gaboxadol; TEAE, treatment-emergent adverse event, TRAE, treatment-related adverse event; SAE, serious adverse event; QD, once daily; BID, twice daily; TID, 3-times daily.

All TEAEs were mild or moderate in severity, with most affected patients experiencing an event of mild severity [overall, 93.8% (15/16); QD, 100% (2/2); BID, 87.5% (7/8); TID, 100% (6/6)]. One-third of all patients (8/23, 34.8%) experienced ≥1 TEAE possibly or probably related to the study medication, with 5/8 (62.5%) of those with any treatment-related TEAE assigned to the OV101 5 mg BID group. No SAE or death was reported. Overall, no trends were observed for hematology, clinical chemistry, and urinalysis laboratory evaluations. There were no notable differences between OV101 treatment groups or discernable trends in vital signs or physical examination changes.

### Efficacy

#### Clinician-Rated Assessments

**FIGURE 3:**
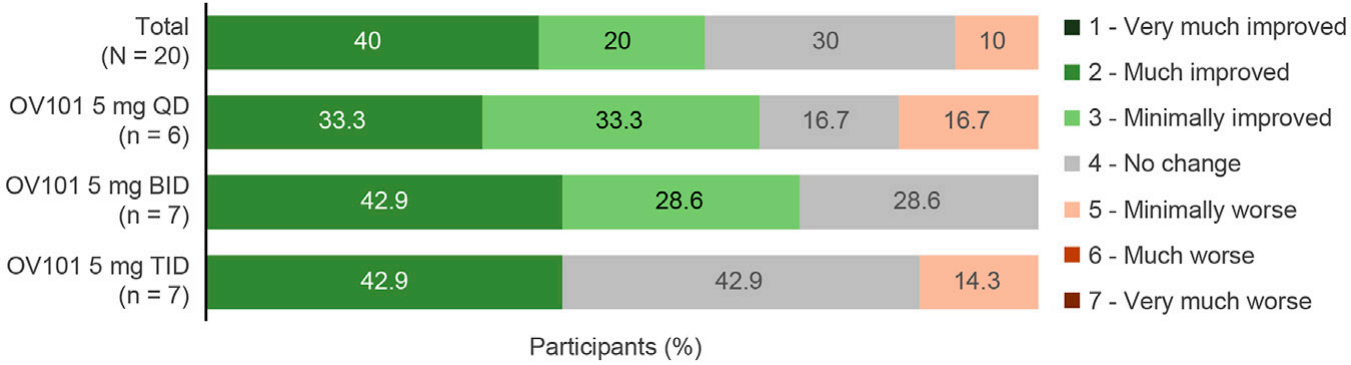
CGI-I score at week 12. Among the 3 dosing regimens, the percentage of CGI-I responders was greatest with OV101 5 mg BID. CGI-I, Clinical Global Impressions–Improvement; OV101, gaboxadol; QD, once daily; BID, twice daily; TID, 3-times daily.

Figure 3 shows that the majority (60.0%) of all OV101-treated participants were rated by clinicians as being CGI-I responders, defined as a score of ≤3 (minimally improved), at week 12, with 40.0% considered “much improved” and 20.0% considered “minimally improved.” The highest percentage of CGI-I responders (71.5%) was observed in the OV101 5 mg BID group, followed by 66.6% in the QD group and 42.9% in the TID group; in the OV101 5 mg BID group, 42.9% of participants were considered “much improved.” Clinician-rated secondary endpoints, such as CGI-S total score (−0.4, *p* < 0.01) and subscale scores related to communication and connectedness (−0.60, *p* < 0.001), anxiety (−0.50, *p* < 0.01), ADHD (−0.5, *p* < 0.05), and VABS-III activities of daily living (ADLs; −0.3, *p* < 0.05), also showed significant improvements from baseline to week 12 (Table 4). No meaningful differences between age groups in clinician-rated assessments were noted (data not shown).

**TABLE 4:** Change in secondary efficacy measures from baseline to week 12.

Mean (SD) score	OV101 5 mg QD (*n* = 7)	OV101 5 mg BID (*n* = 8)	OV101 5 mg TID (*n* = 8)	Total (*n* = 23)
CGI-S	(*n* = 6)	(*n* = 7)	(*n* = 7)	(*n* = 20)
Total score	−0.7 (0.52)	−0.3 (0.49)	−0.3 (0.49)	−0.4 (0.50)
Anxiety	−0.3 (0.82)	−0.6 (0.79)	−0.6 (0.79)	−0.5 (0.76)
ADHD	−1.0 (1.10)	−0.3 (0.76)	−0.1 (0.38)	−0.5 (0.83)
Communication/connectedness	−1.0 (0.89)	−0.6 (0.53)	−0.3 (0.49)	−0.6 (0.68)
Repetitive & restrictive behavior	−0.3 (0.52)	−0.1 (0.38)	0.0 (0.58)	−0.2 (0.49)
Disruptive behavior	−1.2 (2.64)	−0.4 (0.79)	−0.6 (1.13)	−0.7 (1.59)
Activities of daily living	−0.5 (0.84)	−0.3 (0.49)	−0.1 (0.38)	−0.3 (0.57)
ABC-C_FXS_	(*n* = 4)	(*n* = 5)	(*n* = 6)	(*n* = 15)
Total score	−20.0 (22.70)	−8.8 (6.57)	−18.2 (16.17)	−15.5 (15.52)
Irritability	−6.3 (10.21)	−3.0 (4.90)	−3.0 (5.06)	−3.9 (6.37)
Lethargy and social withdrawal	−6.0 (7.16)	−3.2 (1.92)	−5.7 (4.63)	−4.9 (4.62)
Inappropriate speech	−0.3 (2.06)	0.4 (2.61)	−1.7 (1.97)	−0.6 (2.26)
Hyperactivity	−4.0 (4.55)	−1.4 (3.44)	−3.2 (1.94)	−2.8 (3.21)
Stereotypic behavior	−2.3 (3.20)	−0.4 (1.52)	−4.2 (3.54)	−2.4 (3.18)
Social avoidance	−1.3 (2.06)	−1.2 (3.11)	−0.5 (3.83)	−0.9 (3.01)
ADAMS	(*n* = 6)	(*n* = 7)	(*n* = 7)	(*n* = 20)
Total score	−10.5 (12.24)	−4.1 (5.64)	−6.9 (10.48)	−7.0 (9.54)
Manic/hyperactive behavior	−2.5 (2.17)	−1.3 (1.89)	−1.3 (1.89)	−1.7 (1.95)
Depressed mood	−0.2 (3.19)	0.1 (1.21)	−0.3 (3.59)	−0.1 (2.69)
Social Avoidance	−4.5 (2.88)	−0.9 (3.08)	−1.4 (3.69)	−2.2 (3.47)
General Anxiety	−2.8 (4.07)	−1.9 (1.86)	−2.9 (2.85)	−2.5 (2.87)
Obsessive/compulsive behavior	−0.5 (3.15)	−0.1 (1.35)	−1.1 (1.35)	−0.6 (1.98)
RBS-R	(*n* = 6)	(*n* = 7)	(*n* = 7)	(*n* = 20)
Total score	−12.0 (15.89)	−3.6 (12.16)	−2.0 (9.18)	−5.6 (12.61)
Conners 3	(*n* = 6)	(*n* = 3)	(*n* = 6)	(*n* = 15)
Inattention	−2.3 (2.94)	−1.0 (2.00)	−2.3 (2.50)	−2.1 (2.49)
Hyperactivity/impulsivity	−1.0 (3.95)	−0.7 (1.15)	−1.3 (2.16)	−1.1 (2.74)
Short Sensory Profile–2	(*n* = 6)	(*n* = 7)	(*n* = 7)	(*n* = 20)
Total score	−8.5 (16.57)	−3.0 (16.10)	−10.0 (8.66)	−7.1 (13.70)

SD, standard deviation; CGI-S, Clinical Global Impressions–Severity; ADHD, attention deficit hyperactivity disorder; ABC-C_FX_, Aberrant Behavior Checklist–Community factor score for fragile X syndrome; ADAMS, Anxiety, Depression, and Mood Scales; RBS-R, Repetitive Behavior Scale–Revised; OV101, gaboxadol; QD, once daily; BID, twice daily; TID, 3-times daily.

#### Caregiver/LAR Assessments

Changes from baseline to week 12 in secondary efficacy measures are summarized in Table 4. There were significant improvements from baseline to week 12 in caregiver-rated ABC-C_FXS_ total score (26%, *p* < 0.01), indicating improvements in common behavioral problems associated with FXS, and secondary endpoints [lethargy/social withdrawal (38%, *p* < 0.01), hyperactivity (29%, *p* < 0.01), stereotypic behavior (21%, *p* < 0.05), and irritability (20%, *p* < 0.05) subdomains]. Using the original scoring method, ABC-C scores were similar to the ABC-C_FXS_ scoring method. There were significant improvements from baseline to week 12 in caregiver-rated ADAMS total score (−0.7, *p* < 0.01) and secondary endpoints [general anxiety (−2.5, *p* < 0.01), social avoidance (−2.2, *p* < 0.05), and manic/hyperactive (−1.7, *p* < 0.01) subscales]. Other secondary efficacy endpoints, such as RBS-R, Short Sensory Profile-2, and Conners 3, did not show meaningful improvements from baseline. Changes in exploratory measures from baseline to week 12 are reported in Table 5. In an exploratory analysis, an improvement in PGI-I, defined as a PGI-I score of ≤3 (modest improvement) at week 12, was reported by 55.0% of caregivers (QD, 50.0%; BID, 71.4%; TID, 42.9%). The top 3 concerns identified by caregivers *via* a VAS total score varied, but the most frequently cited were related to anxiety, disruptive behavior, and ADLs. Based on a preliminary analysis, there do not seem to be noteworthy differences in sleep behavior based on the sleep CGI and actigraphy (data not shown). No meaningful differences between age groups in caregiver/LAR assessments were noted (data not shown).

**TABLE 5:** Change in exploratory efficacy measures from baseline to week 12.

Mean (SD) score	OV101 5 mg QD (*n* = 7)	OV101 5 mg BID (*n* = 8)	OV101 5 mg TID (*n* = 8)	Total (*n* = 23)
PGI-S total score	−0.7 (0.52)	−0.7 (0.95)	−0.3 (0.76)	−0.6 (0.76)
Caregiver top 3 concerns VAS^a^				
Top concern 1	−1.02 (4.56)	0.93 (1.71)	0.57 (2.40)	0.22 (2.99)
Top concern 2	−0.50 (5.13)	3.49 (2.71)	1.09 (2.46)	1.45 (3.73)
Top concern 3	−1.92 (4.96)	1.14 (1.91)	0.27 (1.27)	0.02 (2.96)
Anxiety	−1.92 (4.96)	0.45 (0.78)	1.33 (2.41)	−0.31 (3.76)
ADHD	0.00 (1.41)	2.27 (2.47)	−0.70 (NA)	1.02 (2.19)
Repetitive/stereotypic behavior	2.00 (2.83)	NA	0.25 (0.35)	1.13 (1.93)
Disruptive behavior	−5.05 (7.00)	0.82 (1.46)	1.05 (4.19)	−0.38 (4.50)
Communication	1.50 (2.12)	5.60 (NA)	0.63 (0.78)	1.59 (2.09)
Activities of daily living	−5.00 (7.07)	0.60 (1.61)	0.23 (1.56)	−0.77 (3.69)
Other	2.00 (2.83)	3.05 (2.81)	0.50 (NA)	2.53 (2.60)

a VAS scoring: 0 (worst behavior) to 10 (best behavior).

SD, standard deviation; PGI-S, Parent Global Impression-Severity; VAS, visual analog scale; ADHD, attention-deficit hyperactivity disorder; OV101, gaboxadol; QD, once daily; BID, twice daily; NA, not available; TID, 3-times daily.

## Discussion

We conducted a 12-weeks, double-blind, parallel-group, phase 2a study of 23 adolescent and adult males with FXS with moderate-to-severe neurobehavioral phenotypes to assess the safety of OV101 and its efficacy in targeting core behavioral symptoms in FXS. By showing OV101 5 mg to be generally safe and well tolerated when administered QD, BID, or TID, this first interventional clinical study of OV101 in FXS achieved its primary outcome. Clinical laboratory tests, electrocardiograms, vital signs, and physical examinations showed no trends or distinct safety signals. The majority of participants (16/23, 69.6%) reported ≥1 dose-dependent TEAE of mild severity (15/16, 93.8%). One-third of participants (8/23, 34.8%) experienced ≥1 TEAE possibly or probably related to the study medication, with 62.5% (5/8) of those with any treatment-related adverse event assigned to the OV101 5 mg BID group. This same group also had mild TEAEs [diarrhea and irritability, 2/23 (9%) each; headache, 3/23 (13%); upper respiratory tract infection, 4/23 (17%)]; and 1 participant who terminated treatment early (moderate agitation). No SAEs or deaths were reported, and there was no evidence of withdrawal effects after the study. Importantly, the favorable safety and tolerability profile of OV101 in this FXS study is consistent with that reported in previous OV101 clinical studies of other conditions (Roth et al., 2010; Bird et al., 2021).

This study also demonstrated an initial efficacy signal for OV101 in FXS based on secondary clinician- and caregiver-rated endpoint outcomes, including CGI-I, CGI-S, ABC-C_FXS_, and ADAMS scores. Using CGI-I as a clinician-rated, syndrome-specific global measure of core features in patients with FXS, the majority of participants treated with OV101 (60%, 12/2) were considered CGI-I responders and 40% (8/20) were rated as “much improved” at week 12. This efficacy signal appeared to be stronger in OV101 5 mg BID and QD groups (71.5 and 66.6% of participants considered CGI-I responders, respectively) versus the TID group (42.9%). From the caregiver perspective, key patient concerns (as captured *via* VAS) were related to anxiety, disruptive behavior, and ADLs. A high rate of anxiety associated with FXS has been previously reported in the literature (Cordeiro et al., 2011; Weber et al., 2019; Budimirovic et al., 2020), which together with social withdrawal (Budimirovic et al., 2006; Kaufmann et al., 2008), can be present in FXS with and without ASD (Cordeiro et al., 2011; Budimirovic and Subramanian, 2016; Niu et al., 2017; Budimirovic et al., 2020). Results of this study showed that OV101 treatment was associated with converging improvements from baseline in maladaptive behaviors at week 12 as assessed by ABC-C_FXS_ (26.2% improvement in total score and improvements in irritability and social withdrawal subscales) and ADAMS (21.6% improvement in total score and improvements in anxiety and social avoidance subscales). Our results also showed an initial signal of improvements in disruptive behavior/ADHD symptoms and ADLs with OV101 as assessed by CGI-S and VABS-III, respectively. Finally, caregiver-rated exploratory analyses of PGI-I at week 12 showed a modest improvement of 55.0%, which again showed the strongest signal in the 5 mg BID (71.4%) versus QD (50.0%) and TID (42.9%) groups. Overall, the initial signal of improvements across multiple domains of the core phenotype of FXS is promising. However, given this is the first clinical study to assess OV101 in FXS, these findings need to be replicated and confirmed in a larger, placebo-controlled study with optimal outcomes and in the most appropriate age group (Duy and Budimirovic, 2017; Berry-Kravis et al., 2018).

The design on this OV101 study had both strengths and limitations that should be considered for future FXS studies. A strength of this study was that it had a sufficiently long duration (12 weeks) to assess behavioral changes related to any potential symptomatic effects of OV101; this is supported by previous studies of autism and other psychiatric conditions in which treatment-derived improvements in behavioral symptoms could be observed within 4 weeks (Berry-Kravis et al., 2018). It also allowed for preliminary evaluation of safety and efficacy at 3 different doses. There were several limitations in the design of this study, the first being the lack of a placebo-control arm. FXS studies have been shown to have large placebo effect for behavioral measures (Berry-Kravis et al., 2018; Luu et al., 2020), with some reporting a placebo effect size similar to the OV101 effect size observed in the present study (Berry-Kravis et al., 2016; Youssef et al., 2018). Another limitation was the small sample size (*n =* 23), which may have been insufficient to assess behavioral efficacy, as prior studies that enrolled more than 100 participants have yielded ambiguous results (Berry-Kravis et al., 2016; Berry-Kravis et al., 2018; Youssef et al., 2018; Berry-Kravis et al., 2020). The size of each treatment arm was small making evaluations of individual OV101 regimens difficult within this study; additional evaluations will be needed to confirm the optimal dose of OV101. Patient stratification is another important factor to consider in FXS studies. Relationships between anxiety and FMRP level and ASD status may help to stratify patients with FXS in clinical studies (Budimirovic et al., 2017; Budimirovic et al., 2020), with results of this study showing that participants taking OV101 5 mg QD or BID achieved greater improvements in problem behaviors then those receiving the highest dosage (5 mg TID). Finally, a separate study is needed to assess the safety, tolerability, and effects of OV101 in female adolescents and adults with FXS.

Given the inherent subjective nature and placebo effects associated with caregiver-rated endpoints (Budimirovic et al., 2017), future FXS studies should also include biomarker endpoints since they can objectively evaluate the efficacy of investigational treatments. The identification and evaluation of valid, sensitive-to-treatment biomarkers is increasingly necessary to reliably track treatment changes in the unfolding wave of clinical studies in FXS (Budimirovic et al., 2017; Erickson et al., 2018; Lee et al., 2018) and to substantially mitigate the large placebo effect in FXS studies. Indeed, an effort to differentiate objective from subjective improvements in individuals with FXS is recommended by experts in the field of FXS (Luu et al., 2020). For example, electroencephalography is a potential neural biomarker sensitive to treatment (Erickson et al., 2018; Ethridge et al., 2019), and molecular studies have shown a link between *FMR1* expansion, gene methylation, and FMRP deficit and the overall severity of the neurobehavioral phenotype (Berry-Kravis et al., 2018; Budimirovic et al., 2020). Newer performance-rated measures, such as expressive language sampling and the NIH Toolbox, may be able to capture real change and better control the placebo effect. Quantification of key receptor expression in the living human brain of men with FXS is also needed to better understand the results of failed FXS clinical studies and to continue to optimize FXS clinical study designs. This measurement may constitute an effective tool to confirm target engagement, for example of NAMs for mGluR5s, in both FXS and ASD (Brašić et al., 2020; Brašić et al., 2021).

In conclusion, the safety, tolerability, and efficacy data from this phase 2a study demonstrate an initial efficacy signal for OV101 in individuals with FXS. The interpretation of the results is confounded by lack of placebo control and small sample size. These results need to be confirmed in a larger, randomized, placebo-controlled study with optimal outcomes and in the most appropriate age group.

## Data Availability

The datasets presented in this article are not readily available because Ovid Therapeutics Inc. is committed to providing qualified scientific researchers appropriate access to anonymized data and clinical study information from the company’s clinical trials for the purpose of conducting legitimate scientific research. Requests for specific data will be considered along with the rationale, description of use need, and clinical value of the proposed analysis. Ovid supports an approach to sharing data that responsibly reflects the interests of all parties involved in clinical trials, including protecting the rights and privacy of trial participants, the innovator’s intellectual property rights, and other incentives for innovation, and as such, will evaluate requests for sharing company clinical trial data with qualified external scientific researchers. Requests to access the data from this clinical trial may be made at clinical@ovidrx.com. Data will be made available for request after product approval in the United States and European Union, after product development is discontinued, or as otherwise required by law or regulation. There are circumstances that may prevent Ovid from sharing the requested data as the product is investigational at this time. Requests to access the datasets should be directed to clinical@ovidrx.com.
